# Effect of Fermented Rapeseed Meal in Feeds for Growing Piglets on Bone Morphological Traits, Mechanical Properties, and Bone Metabolism

**DOI:** 10.3390/ani13061080

**Published:** 2023-03-17

**Authors:** Siemowit Muszyński, Aleksandra Dajnowska, Marcin B. Arciszewski, Halyna Rudyk, Jadwiga Śliwa, Dominika Krakowiak, Małgorzata Piech, Bożena Nowakowicz-Dębek, Anna Czech

**Affiliations:** 1 Department of Biophysics, Faculty of Environmental Biology, University of Life Sciences in Lublin, 20-950 Lublin, Poland; 2 Department of Animal Anatomy and Histology, University of Life Sciences in Lublin, 20-950 Lublin, Polandmb.arciszewski@wp.pl (M.B.A.);; 3 Department of Animal Physiology, Faculty of Veterinary Medicine, University of Life Sciences in Lublin, 20-950 Lublin, Poland; 4 Department of Animal Hygiene and Environmental Hazards, Faculty of Animal Sciences and Bioeconomy, University of Life Sciences in Lublin, 20-950 Lublin, Poland; bozena.nowakowicz@up.lublin.pl; 5 Department of Biochemistry and Toxicology, Faculty of Animal Sciences and Bioeconomy, University of Life Sciences in Lublin, 20-950 Lublin, Poland; anna.czech@up.lublin.pl

**Keywords:** piglets, fermented rapeseed meal, bones, mechanical properties, bone markers

## Abstract

**Simple Summary:**

Strong and healthy bones allow for efficient locomotion and overall functioning of the musculoskeletal system of pigs. This is the first study to provide information on the effect of including fermented rapeseed meal as a partial wheat replacement in the diet on bone quality in weaner piglets.

**Abstract:**

Quality feed is essential for correct bone development and proper functioning of animals. Post-weaned piglets experience a radical change in eating behaviour that can influence their feed intake. For this reason, functional feed additives and ingredients that can be used in post-weaning feeds are needed. The objective of this study was to evaluate the effects of partially replacing wheat with rapeseed meal fermented using *Bacillus subtilis* strain 87Y on overall bone quality and bone metabolism in weaner piglets. From the 28th day of life, barrows were fed either a standard wheat-based diet or a diet containing 8% fermented rapeseed meal (FRSM) with or without a feed additive containing enzymes, antioxidants, probiotics, and prebiotics. The experimental period lasted 60 days, after which femur quality indices were assessed. Differences in bone length and weight were observed, but there were no changes in bone mineralization or bone mid-diaphysis morphometrical traits between treatments. FRSM inclusion reduced bone mid-diaphysis biomechanical properties, but these changes were dependent on feed-additive supplementation. Analysis of the levels of serum bone turnover markers suggests the intensification of bone resorption in FRSM-fed groups as deoxypyridinoline levels increase. The results obtained warrant further research on what the disturbances in bone mechanical properties and metabolism observed in FRSM-fed weaners means for the subsequent fattening period.

## 1. Introduction

Rapeseed meal (RSM) is the by-product of crude rapeseed oil extraction obtained after the pressing process. Since the nutritional value of RSM extract is still high (contains 25–40% digestible proteins), it is routinely used as a good ingredient in the feeds of a variety of livestock [1]. Additionally, more than 83.14 million metric tonnes of rapeseed are produced worldwide [2]. However, besides its relatively good protein composition, some components such as oxazolidinethione, isothiocyanates, thiocyanates, glucosinolate-derived nitriles, tannins and phytic acid, which limit the utilization of basal nutrients, are also present in RSM [3,4]. Feeding trials have shown that the glucosinolates present in rapeseed press cake reduce feed intake and weight gain in experimental pigs [5]. Certain technologies have been developed to reduce the levels of anti-nutritional components and to improve the nutritional value and digestibility of RSM. The most common methods rely on the hydrolyzation catalysed by enzymes [6], as well as on dehulling [7] and fermentation [4,8]. The latter seems to be the method of choice, since it is well known that during fermentation the structure of plant cell walls is decomposed, allowing the production or release of biologically active substances, including antioxidants [9]. Several experiments have demonstrated that fermented RSMs (FRSMs) positively affect intestinal morphology [10,11], gut microflora [12] and immune status [8,13,14,15].

It is well known that quality feed is essential for proper bone development and enables the attainment of a bone mass necessary for the proper functioning of animals [16,17]. Nutritional components such as inorganic minerals are of primary importance and their deficiency usually leads to reduced bone mass, osteoporosis, increased frailty, and fractures. Proteins are also very important because it is generally believed that higher protein intake might be beneficial for bone mass [18], although some authors have found it prudent not to combine a high protein diet with low calcium intake since this combination might lead to increased risk of bone fractures [19]. The primary role of bones is to protect internal organs, ensure motion and counteract gravity. However, taking into account the fact that farm animals are increasingly being bred to have higher muscular mass, bones have to bear greater and greater loads. Measuring bone mechanical properties in experimental studies is a very powerful tool that helps in the evaluation of bone quality [20].

The immunomodulatory properties of fermented components in pig feeds, for example FRSM or fermented soybean meal, limit intestinal diseases and improve homeostasis in the body [11,14], showing beneficial effects on the microbiome and in performance parameters [11,14]. However, how it influences bone metabolism and bone mechanical endurance is not known.

Therefore, the present study evaluated the effect of including FRSM—a protein-rich ingredient—in the diet on weaners’ bone quality when consumed over a 60-day period during the growing phase, after which bone morphological traits, mechanical properties and serum levels of bone turnover markers were assessed. It was also hypothesized that due to its immunomodulatory properties, FRSM can be added to the diets of weaners with or without the inclusion of feed additives (enzymes, organic acids, antioxidants, additional Zn, probiotics and prebiotics).

## 2. Materials and Methods

The study was conducted in accordance with the ARRIVE guidelines. The experimental procedure was approved by the Local Ethics Committee on Animal Experimentation of the University of Life Sciences in Lublin, Poland (approval no. 50/2018, of 1 April 2018).

### 2.1. Preparation of Fermented Rapeseed Meal (FRSM)

FRMS was obtained through fermentation using *Bacillus subtilis* 87Y from the strain collection at InventionBio (Bydgoszcz, Poland). The preparation of bacterial inoculum as well as the procedure for RPS aerobic fermentation through which FRSM was obtained is described in detail in [21]. In brief, resuspended *Bacillus subtilis* 87Y precultures were mixed with pasteurized RSM in a 1:1 ratio to achieve 50% humidity. Solid-state fermentation was performed at 37 °C for 24 h. After fermentation, the obtained biomass was snap frozen at −80 °C and then air-dried. The FRSM composition is presented in Supplementary Table S1 [8].

### 2.2. Animals and Study Design

A total of 144 Yorkshire x Danish Landrace crossbred, clinically healthy barrows weaned at 28 days of age were used in this study. The barrows were individually tagged, weighed and assigned to one of three dietary treatment groups, with equal numbers of barrows in each group. Each dietary treatment consisted of six replicate pens, with 8 barrows per replicate. Body weight variation within and between each pen/replicate and group was minimized as far as practically possible. The barrows were divided into controls (the Contr group), those fed a standard diet, and two experimental groups (FRA and FR groups), which were fed 8% FRSM [8,14]. Additionally, the FR group, in contrast to the FRA group, was fed a diet lacking 0.33% of the feed additive. The same feed additive was included in the feed given to the Contr group. The additive was mixed with the bulk feed and subjected to a technological agglomeration process to produce feed pellets. As the experiment was conducted in an actual pig breeding farm, all diets (Table 1 and Table 2) were formulated to meet or exceed the nutritional requirements specified by the NRC [22], irrespective of additional supplementation with the feed additive. Barrows were fed ad libitum and had free access to water. Prior to the experiment, the animals were examined by a veterinarian to confirm that their health status would not affect the results.

### 2.3. Sample Collection

The experiments were conducted for 60 days. At the end of the experiments, animals were fasted overnight and one barrow chosen randomly from each replicate pen was selected for weighing and blood collection. The animals were then euthanized using i.m. injections of Ketamine (350 mg/100 kg b.w.), Stresnil (200 mg/100 kg b.w.), Sedazin (30 mg/100 kg b.w.) and intravenous Morbital, 26.7 mg/mL (0.3–0.6 mL/kg b.w.). Immediately after euthanasia, the femurs were dissected, cleaned of adherent tissue, weighed, wrapped in gauze soaked in isotonic saline and frozen at −26 °C until analysis. The total number of euthanized animals was 18 (*n* = 6 per treatment group). The left femur was earmarked for densitometry and biomechanical test, while the right femur was used to measure bone mid-diaphysis geometry. Serum samples were prepared by centrifuging coagulated blood (1300× *g* for 10 min). The collected serum was aliquoted and stored at −86 °C until assays were performed.

### 2.4. Bone Analysis

Prior to the analysis, bones were thawed at room temperature for 6 h. Bone density was assessed by determining whole bone mineral content (BMC) and bone mineral density (BMD) using the DXA method (Norland XR 43 densitometer, Norland, Fort Atkinson, WI, USA) and calculating the Seedor index by dividing the weight of the left femur by its length [23]. To evaluate the mechanical properties of bone mid-diaphysis, a 3-point bending test was performed using a universal testing machine (Zwick Z010, Zwick, Ulm, Germany). During the test, the load was applied at a constant rate of 10 mm/min until the bone fractured. Recorded load-deflection curves were used to determine mechanical properties of femurs in the elastic region of deformation (yield force, yield deflection, elastic work and stiffness) and at bone breakage (breaking force, breaking deflection and breaking work). The right femur was cut transversally at the midpoint of the bone diaphysis using a diamond bandsaw (MBS 240/E, Proxxon GmbH, Foehren, Germany) and the external and internal diameters of the mid-diaphysis cross-section of medial-lateral (horizontal) and cranial-caudal (vertical) planes were measured using a digital calliper, enabling the calculation of the following geometric parameters of bone mid-diaphysis cross-section: mean relative wall thickness (MRWT), cortical index, cross-sectional area (CSA), and cross-sectional moment of inertia about medial-lateral axis [24]. Femoral properties (yield strain, yield stress, breaking strain, and breaking stress) were determined on the basis of previously calculated femur mechanical and mid-shaft geometrical parameters [24].

### 2.5. Bone Turnover Markers

Blood serum levels of the following bone turnover markers were quantified using commercial pig-specific enzyme-linked immunosorbent assay (ELISA) kits: C-terminal telopeptide of type I collagen (CTX-I; QY-E40211, Qayee Biotechnology, Shanghai, China), pyridinoline (PYD; QY-E4020, Qayee Biotechnology), deoxypyridinoline (DPD; QY-E40210, Qayee Biotechnology), osteocalcin (OC; QY-E40100, Qayee Biotechnology), receptor activator of nuclear factor κ B ligand (RANKL, AMS.E07R0392, AMS Biotechnology, Abingdon, UK) and osteoprotegerin (OPG; ELK5799, ELK Biotechnology, Wuhan, China). All assays were performed on two technical replicates according to the manufacturers’ protocols using an Epoch microplate spectrophotometer (Agilent, St. Clara, CA, USA).

### 2.6. Statistical Analysis

Data were analysed using Statistica 13 software (TIBCO Software Inc., Palo Alto, CA, USA), with each barrow as the experimental unit. Distribution of the variables was tested for normality using the Shapiro–Wilk test. Normally distributed variables were compared using one-way analysis of variance (one-way ANOVA). When a treatment effect was observed, Tukey’s post hoc test was used to determine the differences between treatments. Where variables were not normally distributed, comparisons were made using the non-parametric Kruskal–Wallis H test and post hoc analysis was conducted using Dunn’s test. For all tests, *p* < 0.05 was taken as statistically significant. Results obtained in each group are presented as mean values and standard error.

## 3. Results

### 3.1. Body Weight

There were no differences in average daily weight gain, daily feed intake or feed efficiency between control and experimental groups throughout the experiment [25].

### 3.2. Basic Femur Properties

**Figure 1:**
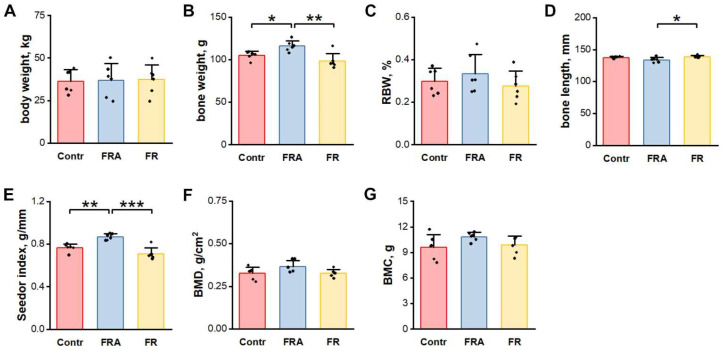
(**A**) Pig weights and basic femur properties of animals in experimental groups: (**B**) bone weight, (**C**) relative bone weight (RBW), (**D**) bone length, (**E**) the Seedor index, (**F**) bone mineral density (BMD), (**G**) bone mineral content (BMC). Bar plots show mean values and standard errors. A range of *p*-values has been assigned above plots where two groups show significant differences: * *p* < 0.05, ** *p* < 0.01, *** *p* < 0.001.

While there were no differences in the mean body weights of pigs in each group (Figure 1A), the femurs of animals fed with FRA were significantly heavier (Figure 1B). However, no differences were observed between treatments when bone weight was normalized to specific animal weight (relative bone weight, RBW) (Figure 1C). The lengths of femurs of pigs in the FRA group decreased significantly in comparison with that of pigs in the FR group (Figure 1D). Among the measured densitometric indices, the Seedor index indicated a significant increase in bone volumetric density in the FRA group (Figure 1E); however, BMD and BMC analyses performed using the DXA method showed that bone mineral content and mineral concentrations were not affected by the inclusion of dietary FRSM (Figure 1F,G).

### 3.3. Geometrical Properties of Femoral Mid-Diaphysis

**Figure 2:**
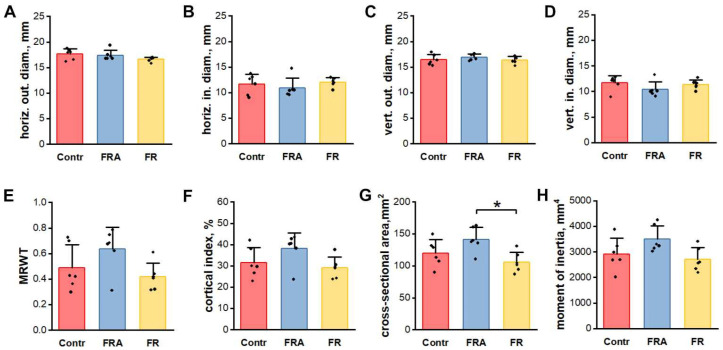
Geometrical properties of bone mid-diaphysis of femurs of animals in experimental groups. (**A**) horizontal external diameter, (**B**) horizontal internal diameter, (**C**) vertical external diameter, (**D**) vertical internal diameter, (**E**) mean relative wall thickness (MRWT), (**F**) cortical index, (**G**) cross-sectional area, (**H**) moment of inertia. Bar plots show mean values and standard errors. A range of *p*-values has been assigned above plots where two groups show significant differences: * *p* < 0.05.

Feeding pigs FRA and FR did not influence femur mid-diaphysis cross-sectional diameters (Figure 2A–D), mean relative wall thickness (MRWT, Figure 2E) or cortical index (Figure 2F), but the femurs of pigs in the FRA group had statistically higher mid-diaphysis cross-sectional areas compared with those of pigs in the FR group (Figure 2G). This difference, however, had no influence on the cross-sectional moment of inertia in the medial-lateral axis (Figure 2G).

### 3.4. Mechanical Properties of Femurs

**Figure 3:**
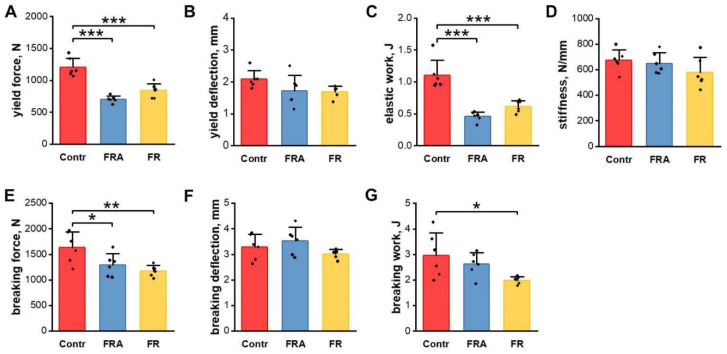
Mechanical properties of bone mid-diaphysis of femurs of animals in experimental groups. (**A**) yield force, (**B**) yield deflection, (**C**) elastic work, (**D**) stiffness, (**E**) breaking force, (**F**) breaking deflection, (**G**) breaking work. Bar plots show mean values and standard errors. A range of *p*-values has been assigned above plots where two groups show significant differences: * *p* < 0.05, ** *p* < 0.01, *** *p* < 0.001.

Dietary FRMS inclusion, both with (the FRA group) and without (the FR group) enzyme supplementation, significantly decreased femur yield force (Figure 3A), elastic work (Figure 3C) and breaking force (Figure 3E). Breaking work of femur in animals in the FR group also significantly decreased compared with those of control animals. On the contrary, dietary treatments had no effect on femur yield and braking deflection (Figure 3B,F, respectively), or on femur bending stiffness (Figure 3D).

### 3.5. Bone Material Properties

**Figure 4:**
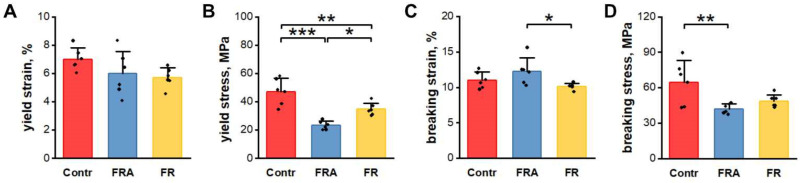
Material properties of bone mid-diaphysis of femurs of animals in experimental groups. (**A**) yield strain, (**B**), yield stress (**C**), breaking strain (**D**), breaking stress. A range of *p*-values has been assigned above plots where two groups show significant differences: * *p* < 0.05, ** *p* < 0.01, *** *p* < 0.001.

While FRMS inclusion had no effect on femur yield strain (Figure 4A), a significant decrease in yield stress was observed in both FRMS-fed groups, with lower yield stress observed in the FRA group (Figure 4B). Breaking strain was significantly higher in the FRA group than in the FR group, although both FRMS-fed groups did not differ from the Contr group (Figure 4C). Significantly lower breaking stress was observed in animals in the FRA group compared with animals in the control group (Figure 4D).

### 3.6. Bone Turnover Markers

**Figure 5:**
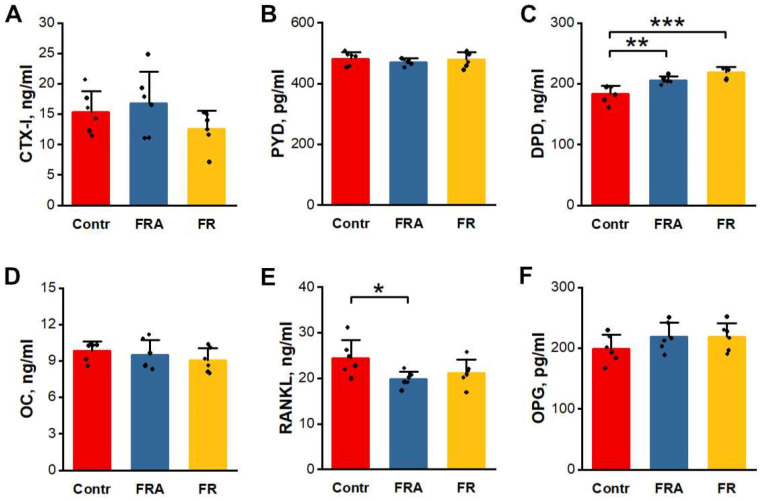
Serum levels of biochemical bone turnover markers in animals in experimental groups. (**A**) C-terminal telopeptide of type I collagen (CTX-I), (**B**) pyridinoline (PYD), (**C**) deoxypyridinoline (DPD), (**D**) osteocalcin (OC), (**E**) receptor activator of nuclear factor κ B ligand (RANKL), (**F**) osteoprotegerin (OPG). A range of *p*-values has been assigned above plots where two groups show significant differences: * *p* < 0.05, ** *p* < 0.01, *** *p* < 0.001.

Feeding pigs FRA and FR did not influence serum levels of markers such as CTX-I (Figure 5A), PYD, (Figure 5B), OC (Figure 5D) and OPG (Figure 5F).

The concentration of DPD was significantly higher in the sera of animals fed either FRA or FR than in the sera of the controls; however, no significant differences were found between the FR and FRA groups (Figure 5C). The level of RANKL in animals fed FRA was significantly lower than in animals in the Contr group, although not in animals in the FR group (Figure 5E).

## 4. Discussion

Until recently, there were limitations to the practical application of introducing rapeseed meal in pigs’ diets because previous studies showed rather negative effects of RSM on animal health and performance due to the presence of numerous anti-nutritional factors [26]. However, numerous studies have shown that fermentation improves the nutritional characteristics of RSM. For example, crude fibres and glucosinolates in FRMS decreased by 25.5% and 43%, respectively [10,27,28,29]. The process of fermentation helps to reduce toxic and anti-nutritional factors present in RSM and improves its quality [3,30,31]. In addition, the microorganisms responsible for the fermentation use the RSM carbohydrate to produce proteins, which are then degraded into peptides [32,33]. Moreover, fermentation increases the quantity of many amino acids [34,35].

Studies have reported that adding 4% or 8% RSM or FRSM, respectively, in the diets of pigs during the growing or finishing phases does not negatively influence growth performance or meat quality, and can even improve their immunological responses [36,37]. A previous study has also shown that the inclusion of 20% solvent-extracted RSM does not affect weight gain; however, this was given for only 28 days [38,39]. Another study also showed that inclusion of 25% RSM in weanling diets had no effect on growth [40]. The inclusion of 30% and 40% RSM was also investigated [41,42], but the period of the study and the age of piglets differed significantly from that presented in the current study. Administering 4% dried FRSM to pregnant sows also improved postnatal bone development in offspring [43].

All these studies evaluated the influence of RSM or FRSM on performance parameters; however, studies on bone metabolism and bone mechanical properties are scarce. Pigs, like many other livestock intended for extensive production systems, are genetically selected to achieve greater weight gain; however, some species are predisposed to bone development disorders, especially under commercially intensive production systems [44]. The bone is a dynamic organ and undergoes two processes, synthesis and resorption, that are kept in balance to maintain normal bone mass necessary for movement and body support, protection of vital organs and general mineral management in the living organism [23,45]. Leg fractures, deformities and bone weakness impair pig welfare and behaviour and are important contributors to economic losses in terms of reduced daily gains and carcass quality [17,46,47,48,49]. Stronger and healthier bones allow for more efficient musculoskeletal performance of the well-supported body. This results in proper behaviour and food intake, thus improving the overall well-being of the pig at all stages of its life.

When studying bone quality in pigs, the metacarpal is generally preferred because it is more appropriate for routine examinations under commercial slaughter conditions [50]. However, due to the fact that no differences in mineralization of various bones are observed in pigs as a result of dietary interventions [50], we selected the femur for our study, as this bone is considered a model bone for biomechanical testing in quadrupedal animals where it is often subjected to bending stress [51].

The present study showed the effect of including 8% FRSM in the diet of pigs in the growth phase on femoral properties. Pigs fed FRSM without enzyme additives have longer bones compared with pigs fed FRSM with feed additives; however, enzyme supplementation resulted in heavier and denser bones (in terms of the Seedor index, a bone volumetric density indicator) with greater cross-sectional area of bone mid-diaphysis. This indicates a positive effect of enzyme supplementation on bone geometry quality indices, as bone weight-bearing capacity, including rapidly growing livestock animals, is associated with bone cross-sectional geometry but not with its length [52]. This indicates that for a certain bone length, pigs whose diets are supplemented with FRSM are able to carry greater weight compared with pigs not fed on FRSM. This is partially in line with other studies showing improved availability of nutritional components in the RSM [11,40,53]. However, the moment of inertia, the geometrical parameter determining the strength of the bone mid-diaphysis against bending loads [54], was not affected by the inclusion of FRSM in the feed. Both cross-sectional area and moment of inertia describe the spatial distribution of bone mass and the observed differences are not necessarily contradictory, as cross-sectional area represents overall bone area, while the moment of inertia describes the distribution of bone mass in relation to the selected axis of the bone, the one in reflection on which the bending test was performed.

The inclusion of FRSM showed no effect on the quantitative indicators of bone mineralization (BMD and BMC). Generally, these indices represent the overall mineral density of bones, which dominates the mechanical functions of bones to a large extent compared with bone volumetric density and ash content [50]. However, although DXA analysis provides measurable insights into bone quality, it estimates the mineral content on the basis of 2D bone scans [55].

Moreover, numerous studies have shown differences in BMD and BMC in different parts of the bone (proximal and distal epiphysis or bone mid-diaphysis) in long bones of pigs [56,57]. Taken together, the possibility that mineralization in some bone regions was affected by dietary treatments cannot be excluded, especially as changes in bone biomechanical parameters were evident in pigs in the FRSM groups.

In both FRSM groups, a reduction in numerous biomechanical parameters, including yield and breaking force, indicate lower bone resistance to breakage despite the lack of changes in femoral mid-diaphysis geometry and mineralization. From a biomechanical point of view, when quantifying differences in bone quality, it is better to discuss bone material properties (relationship between stress and strain), since they correctly measured raw bending or breaking forces, or deflections for bone mass spatial distribution (bone geometry) and bending test procedures (loading rate, span distance) [58,59,60]. The lack of changes in the calculated femur yield strain and increased breaking strain observed in pigs fed FRSM containing enzymes indicated that the bones of these pigs showed greater bone deflection in both plastic regions of deformation compared with pigs in the enzyme-deprived group. Furthermore, the bones of pigs in the FRA group were characterized by the lowest internal stress values at which deformation changed from elastic to plastic (yield stress). To explain changes in the bone characteristics in pigs fed on FRSM, especially those in the FRA group, an analysis of the bone organic phase is required, especially collagen fibres, whose network not only contributes significantly to the elastic properties of bones, but is also responsible for tissue integrity, since it provides a structural scaffold for the mineral phase [51].

The bone mechanical strength results are consistent with measured serum markers of bone homeostasis and turnover rate. In the present study, the concertation of pyridinoline (PYD) and deoxypyridinoline (DPD), the two cross-linking compounds of collagen fibres that stabilize mature collagen derived from an enzymatic pathway initiated by the enzyme lysyl oxidase [61], was identified in the sera of experimental animals. One of these, DPD, increased significantly in pigs fed on FRSM. In addition, the C-terminal telopeptide of fibrillar I collagen (CTX-I) has been used as a biomarker of bone turnover rate. Second, osteocalcin (OC), which is secreted by mature bone cells, is the most abundant non-collagen protein in the bone and is an indicator of bone mineralization. Both markers were not affected by the inclusion of FRSM, while the increase in DPD indicated an intensification of bone resorption. This was especially evident in the FR group, which was deprived of feed additive supplementation containing, among others, additional zinc. It is well known that dietary zinc is essential for collagen synthesis, stimulation of DNA synthesis in osteoblasts and reduction in osteoclast resorption [62,63,64]. On the other hand, bone homeostasis regulates not only growth hormone or insulin-like growth factor, but also the RANK/RANKL/OPG system [65,66,67]. Studies in pigs show the possibility of nutritional modulation of this system [68,69,70,71]. The current study showed that FRSM inclusion in feed for growing pigs intensified bone turnover processes and the imbalance between bone synthesis and resorption, which confirm other findings in the study. All observed changes in bone properties were dependent on the intensification of bone turnover and the activities of osteoblasts and osteoclasts. The main factors involved in this process are OPG, an osteoclastogenesis inhibitor released by osteoblasts, and RANKL, a glycoprotein released by mature osteoblasts and their precursors, that activate the process of osteoclast maturation, next released by osteoclasts themselves. The binding of RANKL to its receptor, RANK (receptor activator of nuclear factor κ B), activates osteoclast precursors that undergo metabolic and structural alteration, leading to bone resorption. OPG, a decoy receptor for RANKL, prevents this process and can inhibit the osteoclast maturation pathway. The maturation, differentiation and metabolic activity of osteoclasts responsible for the intensity of bone resorption depend on the balance between RANKL and OPG. When RANKL levels are higher than OPG levels, bone resorption is increased [72]. As indicated in the current study, the equilibrium between OPG and RANKL levels was not maintained, and this was an important reason for the worsening bone metabolism, additionally supported by markers of bone resorption (increase in DPD levels), which all together worsened bone parameters. There is a need for further investigation of this issue in light of the results obtained.

Impaired bone metabolism in RSM-fed pigs during the production cycle intended for fattening cannot be a reason to stop further research, especially since the lifespan of pigs is relatively short. First, it is important to replace soy in the diet with another source of protein and energy. As mentioned above, rapeseed is a very common crop that is not genetically modified [73]. Moreover, even though some studies show that the weight gained is comparable to or a little lower than that observed with traditional feeding, the feed conversion ratio is significantly improved [4,73]. Furthermore, dietary FRSM can prevent intestinal dysbiosis in weaners [74].

## 5. Conclusions

In conclusion, although it seemed that including 8% FRSM in growing piglets’ diets was not optimal for proper bone mechanical properties and bone turnover, no changes were observed in overall bone mineralization, meaning that FRSM is still a valuable protein and energy source, especially since numerous studies on pigs have shown a lack of negative effects of FRMS on animal performance. Nevertheless, the results obtained warrant further research on what this means for the subsequent fattening period.

## Figures and Tables

**Table 1:** Ingredient composition (% of air-dried matter) and nutrient content of piglets’ diets [14].

Ingredient, %	Group		
	Contr	FRA	FR
Wheat	60.48	58.33	58.8
Barley	20	20	20
Soybean meal, 46.5% CP	9.24	3.46	3.32
Fermented rapeseed meal (FRSM)	0	8	8
Fish meal, 65%	4	4	4
Soybean oil	2.1	2.13	2.13
Chalk 0.95 0.87 0.87	0.95	0.87	0.87
L-Lysine_HCl, 78%	0.82	0.91	0.91
L-Threonine	0.37	0.4	0.4
DL-Methionine	0.26	0.25	0.25
Sodium chloride	0.43	0.39	0.39
Calcium monophosphate	0.52	0.43	0.43
Premix ^1^	0.5	0.5	0.5
Feed additive ^2^	0.33	0.33	0

^1^ Mineral-vitamin premix, content in 1 kg: Ca, 240 g; K, 1 g; Fe, 20 g; Mn, 11 g; Cu, 2.5 g; Se, 60.0 mg; I, 120 mg; Co, 150 mg; vit. A, 1,600,000 IU; vit. D3, 200,000 IU; vit. E, 10.0 g; vit. K, 600 mg; vit. B1, 500 mg; vit. B2, 1400 mg; vit. B6, 800 mg; vit. B1,2 10.0 mg; nicotinic acid, 4.0 g; pantothenic acid, 4 g; chloric choline, 40 g; folic acid, 300 mg. ^2^ Feed additive content in 1 kg feed: mixture of formic and propionic acid 3:1 (2.5 g); *E. coli* phytase (0.15 g with an activity of 5000 FTU/g); xylanase (0.15 g with an activity of 12,200 U/g); beta-glucanase (0.15 g with an activity of 1520 U/g); pentosanase, hemicellulase and enzymes that can hydrolyse pectic substances (0.10 g); ZnO (78%, 0.160 g Zn), *Saccharomyces cerevisiae* (0.20 g).

**Table 2:** Metabolized energy (ME, MJ/kg) and chemical composition (g/kg) of experimental diets [14].

Item	Group		
	Contr	FRA	FR
ME, MJ/kg	13.48	13.51	13.50
Crude protein	170.9	170.2	169.9
Crude fat	37.65	39.05	40.45
Crude fibre	27.14	29.64	30.14
Crude ash	51.83	56.85	51.27
Ca	7.07	7.00	7.14
P	5.47	5.54	5.43
Phytin P	2.47	2.08	2.04
Fe	0.204	0.209	0.205
Cu	0.015	0.016	0.015
Zn	0.167	0.169	0.040
Lactic acid	0.885	5.28	5.00
Tannins	7.13	3.22	4.98
Glucosinolates, mmol/kg	11.40	0.101	0.987

## Data Availability

The data presented in this study are available on request from the corresponding author.

## Supplementary Materials

The following supporting information can be downloaded at: https://www.mdpi.com/article/10.3390/ani13061080/s1, Table S1: Content (g) of analysed nutrients and bioactive substances in 1 kg of FRSM.

## References

[1] Gołębiewska K., Fraś A., Gołębiewski D. (2022). Rapeseed Meal as a Feed Component in Monogastric Animal Nutrition—A Review. Ann. Anim. Sci..

[2] Ates A.M., Bukowski M. Oil Crops Outlook: September 2022. https://www.ers.usda.gov/publications/pub-details/?pubid=104713.

[3] Bell J.M. (1984). Nutrients and Toxicants in Rapeseed Meal: A Review. J. Anim. Sci..

[4] Cheng H., Liu X., Xiao Q., Zhang F., Liu N., Tang L., Wang J., Ma X., Tan B., Chen J. (2022). Rapeseed Meal and Its Application in Pig Diet: A Review. Agriculture.

[5] Schöne F., Tischendorf F., Leiterer M., Hartung H., Bargholz J. (2001). Effects of Rapeseed-press Cake Glucosinolates and Iodine on the Performance, the Thyroid Gland and the Liver Vitamin a Status of Pigs. Arch. Tierernaehrung.

[6] Rodrigues I.M., Carvalho M.G.V.S., Rocha J.M.S. (2014). Increasing the Protein Content of Rapeseed Meal by Enzymatic Hydrolysis of Carbohydrates. Bioresources.

[7] Kracht W., Dänicke S., Kluge H., Keller K., Matzke W., Hennig U., Schumann W. (2004). Effect of Dehulling of Rapeseed on Feed Value and Nutrient Digestibility of Rape Products in Pigs. Arch. Anim. Nutr..

[8] Grela E.R., Czech A., Kiesz M., Wlazło Ł., Nowakowicz-Dębek B. (2019). A Fermented Rapeseed Meal Additive: Effects on Production Performance, Nutrient Digestibility, Colostrum Immunoglobulin Content and Microbial Flora in Sows. Anim. Nutr..

[9] Zhao Y.-S., Eweys A.S., Zhang J.-Y., Zhu Y., Bai J., Darwesh O.M., Zhang H.-B., Xiao X. (2021). Fermentation Affects the Antioxidant Activity of Plant-Based Food Material through the Release and Production of Bioactive Components. Antioxidants.

[10] Hu Y., Wang Y., Li A., Wang Z., Zhang X., Yun T., Qiu L., Yin Y. (2016). Effects of Fermented Rapeseed Meal on Antioxidant Functions, Serum Biochemical Parameters and Intestinal Morphology in Broilers. Food Agric. Immunol..

[11] Czech A., Grela E.R., Kiesz M. (2021). Dietary Fermented Rapeseed or/and Soybean Meal Additives on Performance and Intestinal Health of Piglets. Sci. Rep..

[12] Szmigiel I., Konkol D., Korczyński M., Łukaszewicz M., Krasowska A. (2021). Changes in the Microbial Composition of the Cecum and Histomorphometric Analysis of Its Epithelium in Broilers Fed with Feed Mixture Containing Fermented Rapeseed Meal. Microorganisms.

[13] Wu Z., Chen J., Ahmed Pirzado S., Haile T.H., Cai H., Liu G. (2022). The Effect of Fermented and Raw Rapeseed Meal on the Growth Performance, Immune Status and Intestinal Morphology of Broiler Chickens. J. Anim. Physiol. Anim. Nutr..

[14] Czech A., Nowakowicz-Debek B., Łukaszewicz M., Florek M., Ossowski M., Wlazło Ł. (2022). Effect of Fermented Rapeseed Meal in the Mixture for Growing Pigs on the Gastrointestinal Tract, Antioxidant Status, and Immune Response. Sci. Rep..

[15] Czech A., Stępniowska A., Kiesz M. (2022). Effect of Fermented Rapeseed Meal as a Feed Component on the Redox and Immune System of Pregnant Sows and Their Offspring. Ann. Anim. Sci..

[16] Palacios C. (2006). The Role of Nutrients in Bone Health, from A to Z. Crit. Rev. Food Sci. Nutr..

[17] Sobol M., Skiba G., Raj S., Kowalczyk P., Kramkowski K., Świątkiewicz M., Grela E.R. (2022). Chemical Body Composition and Bone Growth of Young Pigs as Affected by Deficiency, Adequate and Excess of Dietary Phosphorus Supply. Ann. Anim. Sci..

[18] Heaney R.P., Layman D.K. (2008). Amount and Type of Protein Influences Bone Health. Am. J. Clin. Nutr..

[19] Bonjour J.-P. (2011). Protein Intake and Bone Health. Int. J. Vitam. Nutr. Res..

[20] Morgan E.F., Unnikrisnan G.U., Hussein A.I. (2018). Bone Mechanical Properties in Healthy and Diseased States. Annu. Rev. Biomed. Eng..

[21] Szmigiel I., Kwiatkowska D., Łukaszewicz M., Krasowska A. (2021). Xylan Decomposition in Plant Cell Walls as an Inducer of Surfactin Synthesis by *Bacillus subtilis*. Biomolecules.

[22] National Research Council (2012). Nutrient Requirements of Swine.

[23] Skomorucha I., Sosnówka-Czajka E. (2021). A Comparison of Morphometric Indices, Mineralization Level of Long Bones and Selected Blood Parameters in Hens of Three Breeds. Ann. Anim. Sci..

[24] Muszyński S., Kwiecień M., Tomaszewska E., Świetlicka I., Dobrowolski P., Kasperek K., Jeżewska-Witkowska G. (2017). Effect of Caponization on Performance and Quality Characteristics of Long Bones in Polbar Chickens. Poult. Sci..

[25] Czech A., Wlazło Ł., Łukaszewicz M., Florek M., Nowakowicz-Dębek B. (2023). Fermented Rapeseed Meal Enhances the Digestibility of Protein and Macro- and Microminerals and Improves the Performance of Weaner Pigs.

[26] Mejicanos G., Sanjayan N., Kim I.H., Nyachoti C.M. (2016). Recent Advances in Canola Meal Utilization in Swine Nutrition. J. Anim. Sci. Technol..

[27] El-Batal A.I., Abdel Karem H. (2001). Phytase Production and Phytic Acid Reduction in Rapeseed Meal by *Aspergillus Niger* during Solid State Fermentation. Food Res. Int..

[28] Pal Vig A., Walia A. (2001). Beneficial Effects of *Rhizopus Oligosporus* Fermentation on Reduction of Glucosinolates, Fibre and Phytic Acid in Rapeseed (*Brassica Napus*) Meal. Bioresour. Technol..

[29] Rozan P., Villaum C., Bau H.M., Schwertz A., Nicolas J.P., Mejean L. (1996). Detoxication of Rapeseed Meal by Rhizopus Oligosporus Sp-T3: A First Step towards Rapeseed Protein Concentrate. Int. J. Food Sci. Technol..

[30] Hill R. (1979). A Review of the ‘Toxic’ Effects of Rapeseed Meals with Observations on Meal from Improved Varieties. Br. Vet. J..

[31] Rakariyatham N., Sakorn P. (2002). Biodegradation of Glucosinolates in Brown Mustard Seed Meal (*Brassica Juncea*) by *Aspergillus* Sp. NR-4201 in Liquid and Solid-State Cultures. Biodegradation.

[32] Hong K.-J., Lee C.-H., Kim S.W. (2004). Aspergillus Oryzae GB-107 Fermentation Improves Nutritional Quality of Food Soybeans and Feed Soybean Meals. J. Med. Food.

[33] Hölker U., Höfer M., Lenz J. (2004). Biotechnological Advantages of Laboratory-Scale Solid-State Fermentation with Fungi. Appl. Microbiol. Biotechnol..

[34] Ouoba L.I.I., Rechinger K.B., Barkholt V., Diawara B., Traore A.S., Jakobsen M. (2003). Degradation of Proteins during the Fermentation of African Locust Bean (*Parkia biglobosa*) by Strains of *Bacillus subtilis* and *Bacillus Pumilus* for Production of Soumbala. J. Appl. Microbiol..

[35] Zhang S.B., Wang Z., Xu S.Y. (2007). Downstream Processes for Aqueous Enzymatic Extraction of Rapeseed Oil and Protein Hydrolysates. J. Am. Oil Chem. Soc..

[36] Yun H.M., Lei X.J., Lee S.I., Kim I.H. (2018). Rapeseed Meal and Canola Meal Can Partially Replace Soybean Meal as a Protein Source in Finishing Pigs. J. Appl. Anim. Res..

[37] Czech A., Grela E.R., Nowakowicz-Dębek B., Wlazło Ł. (2021). The Effects of a Fermented Rapeseed Meal or/and Soybean Meal Additive on the Blood Lipid Profile and Immune Parameters of Piglets and on Minerals in Their Blood and Bone. PLoS ONE.

[38] Landero J.L., Beltranena E., Cervantes M., Morales A., Zijlstra R.T. (2011). The Effect of Feeding Solvent-Extracted Canola Meal on Growth Performance and Diet Nutrient Digestibility in Weaned Pigs. Anim. Feed Sci. Technol..

[39] Landero J.L., Wang L.F., Beltranena E., Bench C.J., Zijlstra R.T. (2018). Feed Preference of Weaned Pigs Fed Diets Containing Soybean Meal, Brassica Napus Canola Meal, or Brassica Juncea Canola Meal. J. Anim. Sci..

[40] Sanjayan N., Heo J.M., Nyachoti C.M. (2014). Nutrient Digestibility and Growth Performance of Pigs Fed Diets with Different Levels of Canola Meal from Brassica Napus Black and Brassica Juncea Yellow1. J. Anim. Sci..

[41] Hong J., Ndou S.P., Adams S., Scaria J., Woyengo T.A. (2020). Canola Meal in Nursery Pig Diets: Growth Performance and Gut Health. J. Anim. Sci..

[42] Parr C.K., Liu Y., Parsons C.M., Stein H.H. (2015). Effects of High-Protein or Conventional Canola Meal on Growth Performance, Organ Weights, Bone Ash, and Blood Characteristics of Weanling Pigs. J. Anim. Sci..

[43] Tomaszewska E., Muszyński S., Dobrowolski P., Kamiński D., Czech A., Grela E.R., Wiącek D., Tomczyk-Warunek A. (2019). Dried Fermented Post-Extraction Rapeseed Meal given to Sows as an Alternative Protein Source for Soybean Meal during Pregnancy Improves Bone Development of Their Offspring. Livest. Sci..

[44] Śliwa E., Radzki R.P., Puzio I. (1996). Osteochondrosis and Tibial Dyschondroplasia in Chickens, Pigs and Foals. Med. Weter..

[45] Upadhaya S.D., Kim I.H. (2020). Importance of Micronutrients in Bone Health of Monogastric Animals and Techniques to Improve the Bioavailability of Micronutrient Supplements—A Review. Asian-Australas. J. Anim. Sci..

[46] van Grevenhof E.M., Heuven H.C.M., van Weeren P.R., Bijma P. (2012). The Relationship between Growth and Osteochondrosis in Specific Joints in Pigs. Livest. Sci..

[47] Gerlinger C., Oster M., Reyer H., Polley C., Vollmar B., Muráni E., Wimmers K., Wolf P. (2021). Effects of Excessive or Restricted Phosphorus and Calcium Intake during Early Life on Markers of Bone Architecture and Composition in Pigs. J. Anim. Physiol. Anim. Nutr..

[48] Lorenzett M.P., Cecco B.S., Bianchi M.V., Cruz R.A.S., Linhares D.C.L., Guedes R.M.C., Driemeier D., Pavarini S.P. (2022). Osteoporosis in Swine. Pesqui. Veterinária Bras..

[49] Crenshaw T.D., Rortvedt-Amundson L.A. Nutritionally Induced Cellular Signals That Affect Skeletal Integrity in Swine. Proceedings of the 23rd International Pig Veterinary Congress (IPVS).

[50] Miesorski M., Gerlinger C., Borgelt L., Lieboldt M.A., Oster M., Wimmers K., Wolf P. Bone Mineralization as Diagnostic Parameter for the Assessment of Dietary Phosphorous Supply in Pigs—Are There Differences between Bones. Proceedings of the 22nd Congress of the European Society of Veterinary and Comparative Nutrition.

[51] Hart N.H., Nimphius S., Rantalainen T., Ireland A., Siafarikas A., Newton R.U. (2017). Mechanical Basis of Bone Strength: Influence of Bone Material, Bone Structure and Muscle Action. J. Musculoskelet. Neuronal Interact..

[52] Lieberman D.E., Polk J.D., Demes B. (2004). Predicting Long Bone Loading from Cross-Sectional Geometry. Am. J. Phys. Anthropol..

[53] Fang Z.F., Peng J., Tang T.J., Liu Z.L., Dai J.J., Jin L.Z. (2007). Xylanase Supplementation Improved Digestibility and Performance of Growing Pigs Fed Chinese Double-Low Rapeseed Meal Inclusion Diets: In Vitro and In Vivo Studies. Asian-Australas. J. Anim. Sci..

[54] Stover K.K., Weinreich D.M., Roberts T.J., Brainerd E.L. (2018). Patterns of Musculoskeletal Growth and Dimensional Changes Associated with Selection and Developmental Plasticity in Domestic and Wild Strain Turkeys. Ecol. Evol..

[55] Muszyński S., Arczewska M., Świątkiewicz S., Arczewska-Włosek A., Dobrowolski P., Świetlicka I., Hułas-Stasiak M., Blicharski T., Donaldson J., Schwarz T. (2020). The Effect of Dietary Rye Inclusion and Xylanase Supplementation on Structural Organization of Bone Constitutive Phases in Laying Hens Fed a Wheat-Corn Diet. Animals.

[56] Sørensen K.U., Shiguetomi-Medina J.M., Poulsen H.D. (2019). Mineralisation of Tubular Bones Is Affected Differently by Low Phosphorus Supply in Growing-finishing Pigs. J. Sci. Food Agric..

[57] Pointillart A., Coxam V., Sève B., Colin C., Lacroix C.H. (2002). Availability of Calcium from Skim Milk, Calcium Sulfate and Calcium Carbonate for Bone Mineralization in Pigs. Reprod. Nutr. Dev..

[58] Crenshaw T.D., Peo E.R., Lewis A.J., Moser B.D., Olson D. (1981). Influence of Age, Sex and Calcium and Phosphorus Levels on the Mechanical Properties of Various Bones in Swine. J. Anim. Sci..

[59] van der Meulen M.C.H., Jepsen K.J., Mikić B. (2001). Understanding Bone Strength: Size Isn’t Everything. Bone.

[60] Ferretti J.L., Capozza R.F., Mondelo N., Zanchetta J.R. (2009). Interrelationships between Densitometric, Geometric, and Mechanical Properties of Rat Femora: Inferences Concerning Mechanical Regulation of Bone Modeling. J. Bone Miner. Res..

[61] McPherson R., Pincus M. (2021). Henry’s Clinical Diagnosis and Management by Laboratory Methods.

[62] Yamaguchi M. (2007). Role of Zinc in Bone Metabolism and Preventive Effect on Bone Disorder. Biomed Res Trace Elements.

[63] Ma Z.J., Yamaguchi M. (2000). Alternation in Bone Components with Increasing Age of Newborn Rats: Role of Zinc in Bone Growth. J. Bone Miner. Metab..

[64] Hadley K.B., Newman S.M., Hunt J.R. (2010). Dietary Zinc Reduces Osteoclast Resorption Activities and Increases Markers of Osteoblast Differentiation, Matrix Maturation, and Mineralization in the Long Bones of Growing Rats. J. Nutr. Biochem..

[65] Dallas S.L., Prideaux M., Bonewald L.F. (2013). The Osteocyte: An Endocrine Cell … and More. Endocr. Rev..

[66] Neve A., Corrado A., Cantatore F.P. (2011). Osteoblast Physiology in Normal and Pathological Conditions. Cell Tissue Res..

[67] Skiba G., Raj S., Sobol M., Kowalczyk P., Grela E.R. (2021). Role of Polyphenols in the Metabolism of the Skeletal System in Humans and Animals—A Review. Ann. Anim. Sci..

[68] Tomaszewska E., Dobrowolski P., Bieńko M., Prost Ł., Szymańczyk S., Zdybel A. (2015). Effects of 2-Oxoglutaric Acid on Bone Morphometry, Densitometry, Mechanics, and Immunohistochemistry in 9-Month-Old Boars with Prenatal Dexamethasone-Induced Osteopenia. Connect. Tissue Res..

[69] Tomaszewska E., Donaldson J., Kosiński J., Dobrowolski P., Tomczyk-Warunek A., Hułas-Stasiak M., Lamorski K., Laskowska-Woźniak D., Muszyński S., Blicharski R. (2021). β-Hydroxy-β-Methylbutyrate (HMB) Supplementation Prevents Bone Loss during Pregnancy—Novel Evidence from a Spiny Mouse (*Acomys Cahirinus*) Model. Int. J. Mol. Sci..

[70] Blicharski T., Tomaszewska E., Dobrowolski P., Hułas-Stasiak M., Muszyński S. (2017). A Metabolite of Leucine (β-Hydroxy-β-Methylbutyrate) given to Sows during Pregnancy Alters Bone Development of Their Newborn Offspring by Hormonal Modulation. PLoS ONE.

[71] Tomaszewska E., Muszyński S., Dobrowolski P., Wiącek D., Tomczyk-Warunek A., Świetlicka I., Pierzynowski S.G. (2019). Maternal HMB Treatment Affects Bone and Hyaline Cartilage Development in Their Weaned Piglets via the Leptin/Osteoprotegerin System. J. Anim. Physiol. Anim. Nutr..

[72] Hofbauer L.C., Kühne C.A., Viereck V. (2004). The OPG/RANKL/RANK System in Metabolic Bone Diseases. J. Musculoskelet. Neuronal Interact..

[73] Skugor A., Kjos N.P., Sundaram A.Y.M., Mydland L.T., Ånestad R., Tauson A.-H., Øverland M. (2019). Effects of Long-Term Feeding of Rapeseed Meal on Skeletal Muscle Transcriptome, Production Efficiency and Meat Quality Traits in Norwegian Landrace Growing-Finishing Pigs. PLoS ONE.

[74] Wlazło Ł., Nowakowic-Dębek B., Ossowski M., Łukaszewicz M., Czech A. (2022). Effect of Fermented Rapeseed Meal in Diets for Piglets on Blood Biochemical Parameters and the Microbial Composition of the Feed and Faeces. Animals.

