# Supplementary material for: Effect of Fermented Rapeseed Meal in Feeds for Growing Piglets on Bone Morphological Traits, Mechanical Properties, and Bone Metabolism
Source: Animals (Basel). 2023 Mar 17;13(6):1080. doi: 10.3390/ani13061080 (PMC10044281; doi:10.3390/ani13061080)
Supplement: Supplementary file 1 [file animals-13-01080-s001.zip › animals-2292538-SI.pdf]

Supplementary Table S1. Content (g) of analysed nutrients and bioactive substances in 1 kg of FRSM [15].

**Item**	FRSM
Metabolizable energy, MJ/kg	12.27
Dry matter	882.7
Crude ash	78.9
Crude protein	291.8
Ether extract	31.7
Crude fiber	91.5
Total P	9.09
Phytin P	5.73
Ca	8.05
Glucosinolates, mmol/kg	11.40
Tannin, g/kg	4.76
Lactic acid, g/kg	50.42
